# Video Capsule Endoscopy in Patients with Chronic Abdominal Pain with or without Associated Symptoms: A Retrospective Study

**DOI:** 10.1371/journal.pone.0126509

**Published:** 2015-04-20

**Authors:** Jeremy Egnatios, Khushboo Kaushal, Denise Kalmaz, Amir Zarrinpar

**Affiliations:** 1 School of Medicine, University of California San Diego, La Jolla, California, United States of America; 2 Department of Medicine, University of California San Diego, La Jolla, California, United States of America; 3 Division of Gastroenterology, University of California, San Diego, La Jolla, California, United States of America; Uppsala University, SWEDEN

## Abstract

**Background:**

Chronic abdominal pain (CAP) is a common indication for gastroenterology referrals. More insidious causes of CAP isolated to the small bowel, such as malignancies and Crohn’s disease, are rising in incidence and causing more gastroenterologists to evaluate their patients with video capsule endoscopy (VCE). However, the role of VCE in patients with CAP is still unclear.

**Aims:**

We assessed the efficacy of VCE in patients with CAP and whether it led to findings that contributed to disease management and meaningful interventions.

**Methods:**

This retrospective study evaluated 607 capsule endoscopy studies at an open referral endoscopy unit. Ninety of the studies were for CAP. These studies were compared to those performed for other indications to compare diagnostic yield. In addition, we investigated whether VCE led to an intervention that improved clinical outcomes.

**Results:**

Overall, the number of abnormal findings in CAP patients was significantly lower than VCE performed for other indications (24.4% vs 39.0%, respectively p = 0.009). When patients with CAP presented with other pertinent clinical findings (e.g. nausea, weight loss, anemia, history of in inflammatory bowel disease, etc.), the likelihood of an abnormal finding increased to a level that was not different from those who received VCE for other indications (27.1%, p = 0.10). The findings from VCE lead to changed management and improved outcomes in 16.2% of CAP patients with associated symptoms. However, the subgroup that benefited the most were those who had a prior history of Crohn’s disease. Patients with CAP who did not have any associated symptoms continued to have a significantly lower abnormal finding rate compared to those who received VCE for other indications (19.4%, p = 0.03) and VCE rarely led to a change in management that would improve outcomes (5.6%).

**Conclusions:**

VCE for CAP has a lower rate of abnormal findings than other indications. However, VCE is a useful diagnostic tool that can help provide a possible etiology of CAP in patients with associated symptoms. However, a change in management from VCE is likely to be limited to those with a history of Crohn’s disease.

## Introduction

Chronic abdominal pain (CAP) is a common gastrointestinal complaint, with a prevalence of up to 21% in the general population [1–3]. It is a significant economic burden, with up to $20 billion in direct and indirect costs [4]. CAP is the 8^th^ most common complaint of all patients presenting in primary care for a health issue and one of the most common reasons for gastroenterology clinic visits [5, 6]. There are many etiologies for abdominal pain ranging from benign and functional disorders to malignancy and inflammatory diseases (IBD), making evaluation of these patients at times difficult. To date, there are no consensus recommendations or practice guidelines for the evaluation of CAP in adults.

Although esophagogastroduodenoscopy (EGD) and colonoscopy are commonly used to evaluate patients with CAP, the role of video capsule endoscopy (VCE) remains unclear. Studies evaluating the diagnostic yield of VCE in CAP patients are limited and inconsistent, with a great range in diagnostic yield (4–44%) [7, 8] and were almost exclusively done in tertiary care referral centers [9]. The studies rarely report whether VCE changed management or improved outcomes. Few studies report how the CAP population are different, both in demographics and their diagnostic yield, from those who received VCE for other reasons. Given the wide range in diagnostic yield, relative safety, and coverage by insurance providers, VCE remains commonly used in practice for the evaluation of patients with CAP [9].

A recent systematic review of studies evaluating the diagnostic potential of VCE in CAP concluded that its use is unclear and conflicting [9]. It also concluded that the current literature regarding the usefulness of VCE in CAP is incomplete considering differences in study parameters, inclusion/exclusion criteria, and varying definitions of outcome reporting. Other factors including different referral base, sample size, and patient characteristics vary greatly between studies. Hence, they limit the interpretation of studies as a whole and obscure the usefulness of performing VCE to evaluate patients with CAP. Finally, the most important aspect of using VCE in practice, whether a diagnosis affected management which improved outcome, is not reported in any previous study.

In this study, we present a retrospective study from the largest cohort of American patients. We investigated the use of VCE in patients with CAP referred to an open endoscopy center. These referrals included those by community physicians as well as from a tertiary referral center. The performance of VCE for CAP was compared with those who received VCE for other indications and the outcomes after diagnosis were also measured. We aimed to determine the yield of performing VCE in CAP and whether it significantly altered management.

## Methods

### Participants

The University of California, San Diego (UCSD) Human Research Protections Program Institutional Review Board approved this retrospective study. Since this was a retrospective study where patient-identifying information was not part of final analysis, no consent was required. UCSD Medical Center Advanced Endoscopy Unit is an open referral center, drawing patients from throughout San Diego and Imperial Counties. Between June of 2004 and July of 2013, 607 capsule endoscopy studies were performed. Studies were examined for primary and ancillary indications, stated by physician, as reason for referral. Patients were excluded if study evaluator determined it incomplete because of poor bowel prep, or if the capsule did not reach the cecum. The endoscopy referral center was not responsible for the workup of CAP and the referral center did not exclude patients who may have had incomplete diagnostic procedures prior to capsule studies. However, almost all (>99%) of the patients had received colonoscopy and EGD studies prior to VCE.

**Fig 1:**
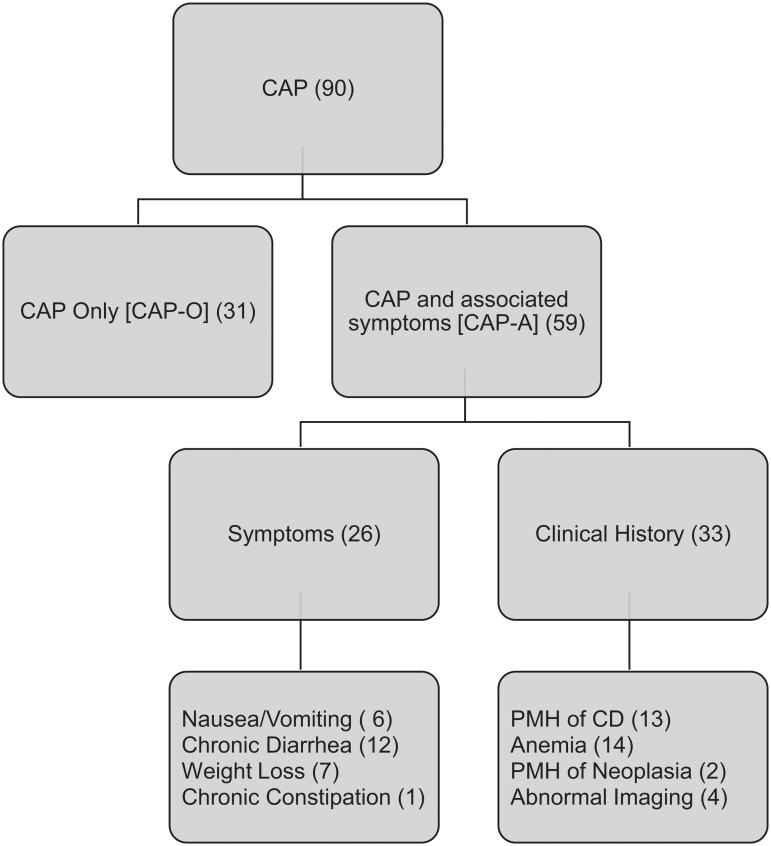
Flowchart with classification of patients with chronic abdominal pain (CAP), along with sub-group classification based on clinical presentation. Abbreviations: PMH (past medical history), CD (Crohn’s disease), UC (ulcerative colitis).

CAP was defined as any type of abdominal pain lasting greater than three months from onset. Patients with CAP were initially evaluated as a single group. With further analysis, they were allocated by their associated symptoms or relevant past medical history (Fig 1). Patients were divided into two groups: CAP only (CAP-O; when no other symptoms or relevant PMH was listed), CAP with associated findings (CAP-A; patients had accompanying chronic nausea, vomiting, diarrhea, weight loss, gastrointestinal bleeding or past medical history of Crohn’s disease, ulcerative colitis, or malignancy). In the CAP-A group, thirteen patients had a past medical history of Crohn’s. We analyzed whether the patients with Crohn’s disease affected group characteristics and later analysis. All analysis was compared with and without the Crohn’s disease patients, with any significant change in findings noted in the results. All remaining non-CAP indications were grouped for control comparison.

### Retrospective Review

All patient information was taken, retrospectively, from the UCSD electronic medical records. When available, patient data collected included: age, sex, body mass index (BMI), past medical history, relevant associated symptoms (such as diarrhea, weight loss, vomiting, etc.), current medications, indication for VCE studies, gastric and small bowel transit times, inpatient status, diagnostic findings, and patient outcome from diagnostic findings. All VCE studies were read by at least two independent gastroenterologists, including one who is highly experienced in small bowel disease and evaluation of small bowel pathology (DK). The gastroenterologists who read the studies were not blinded to the reason for referral. Studies were considered to be positive for an abnormal finding if any imaging deemed abnormal by endoscopist was in the report, regardless of whether the observed findings could be related to patient’s presenting symptoms.

Additional chart review was performed on patients with abnormal finding. Differences in demographics, referral source, and serum inflammatory markers were assessed. Their records were then evaluated for follow up and management for at least 6 months after VCE. We assessed whether successful interventions or disease management were a result of abnormal findings from VCE. Patient outcomes were determined by reviewing physician’s notes in the patient’s chart.

### Sensitivity Analysis

In order to investigate whether the benefit from interventions for VCE was comparable to that observed for other indications of VCE (i.e. GI bleed), we performed a sensitivity analysis. Patients who received a VCE for obscure gastrointestinal bleeding (VCE-GIB patients) were used as a separate cohort for this analysis. This subset of patients was then categorized by age and gender with no other information available to the investigator. Each patient with CAP was then matched to VCE-GIB patients in a 1:2 ratio (that is 2 VCE-GIB patients for each 1 CAP patient) for all patients that had follow up. Hence we made two separate cohorts, VCE-GIB-mA, who were matched to the CAP-A patients, and VCE-GIB-mO, who were matched to the CAP-O patients. As before, chart review for at least six months following VCE study was performed on the VCE-GIB patients and any information on intervention, further study, outcome, or resolution was recorded.

### Statistical Analysis

Statistical analysis was performed using the Statistical Package for Social Science (SPSS) version 20.0 (SPSS, Inc., Chicago, Il) and GraphPad Prism 5 (GraphPad Software, La Jolla, CA). Student t-test was used to compare continuous variables (e.g. age, BMI, etc.). Chi-square analysis was used to compare categorical variables (e.g. abnormal findings, interventions, gender). Given the limited availability of VCE specialists and the referrals seen in this study, intent to treat analysis was applied to all comparisons, as we felt this best evaluated the true clinical impact of VCE. For all of statistical tests, an alpha level of less than 0.05 (two-tailed) was set for significance.

## Results

### Demographics

During the nine year period of the study, 607 capsule studies were performed, with 510 meeting the inclusion criteria. The majority of excluded studies, 85 out of 97 (88%), were due to the capsule not reaching the cecum. Ninety studies were for CAP, with 31 performed in patients who did not have any other symptoms. The remaining 59 studies were performed in patients who complained of CAP along with other associated symptoms (Fig 1). Patients who received VCE for other indications (e.g. iron deficiency anemia, overt bleeding, polyposis syndrome, etc.) were grouped into one cohort as a control for comparison (non-CAP; S1 Fig). There were demographic differences between patients with CAP and those in our control, non-CAP cohort (Table 1). Patients with CAP were significantly younger (43.7 years vs 55.5 years, p = 3.0 x 10^-5^) and more likely to be women, (66.7% v. 51.0%, p = 0.001). None of the patients in the CAP group had a retained capsule or other complication from VCE.

**Table 1:** Characteristics of patients with CAP and those receiving VCE for all other indications.

	CAP			Non-CAP	
	CAP-O(n = 31)	CAP-(n = 59)	Total (n = 90)	Total (n = 420)	p-value*
**Age (yr)**	43.1 (±2.3)	44.6 (±2.7)	43.7 (±1.7)	55.5 (±0.93)	0.00003^a^
**Gender (%)**	58.1%	71.2%	66.7%	51.0%	0.001^b^
**BMI (kg/m^2^)**	24.0 (±0.8)	24.5 (±0.5)	24.1 (±1.0)	26.5 (±0.5)	0.03^a^
**% Inpatient**	3.1%	19%	13.2%	19.1%	0.2^b^
**% Abnormal Finding**	19.4%	27.1%	24.4%	39.0%	0.009^b^

CAP-O represents CAP with no other symptoms. CAP-A represents CAP and any associated symptoms (See Methods for description).

*P value represents analysis between all patients with CAP and Non CAP groups.

^a^Student t test.

^b^χ^2^ test.

### Abnormal Finding Rate

**Fig 2:**
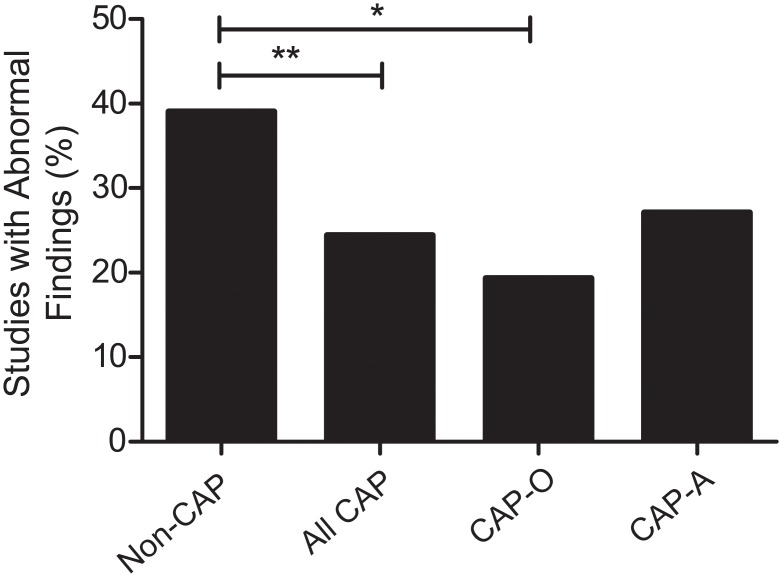
Abnormal findings in those receiving VCE for CAP versus those receiving VCE studies for all other indications (non-CAP). CAP-O represents CAP with no other symptoms. CAP-A represents CAP and any associated symptoms (See Methods for description). * represents a p-value of < 0.05. ** represents a p-value of < 0.01

VCE studies performed for CAP were less likely to yield an abnormal finding compared to the control group, (24.4% vs 39.0%, respectively, p = 0.009). Since the patients with CAP are a heterogeneous group, we subdivided them into two groups: (1) CAP only (CAP-O, 31 patients) and (2) CAP with associated symptoms (CAP-A, 59 patients) (see methods for description of each group; Fig 1). On additional investigation of clinical presentation of CAP, no significant differences were found in rates of abnormal findings between the two CAP subgroups (19.4% for CAP-O; 27.1% for CAP-A; Fig 2). However, only the CAP-O group had significantly lower abnormal yield than the control group (p = 0.02).

Since other symptoms appeared to affect the rate of abnormal findings, we investigated whether more objective measures, such as serum biomarkers, would as well. Specifically, inflammatory markers, such as CRP, ESR, and leukocytosis, as well as rheumatological markers such as ANA were analyzed for patients with a positive abnormal finding on VCE and those without any abnormal findings on VCE. However, none of these serum makers were predictive of an abnormal finding in CAP patients (S1 Table).

### Community versus Tertiary Care Referral

**Fig 3:**
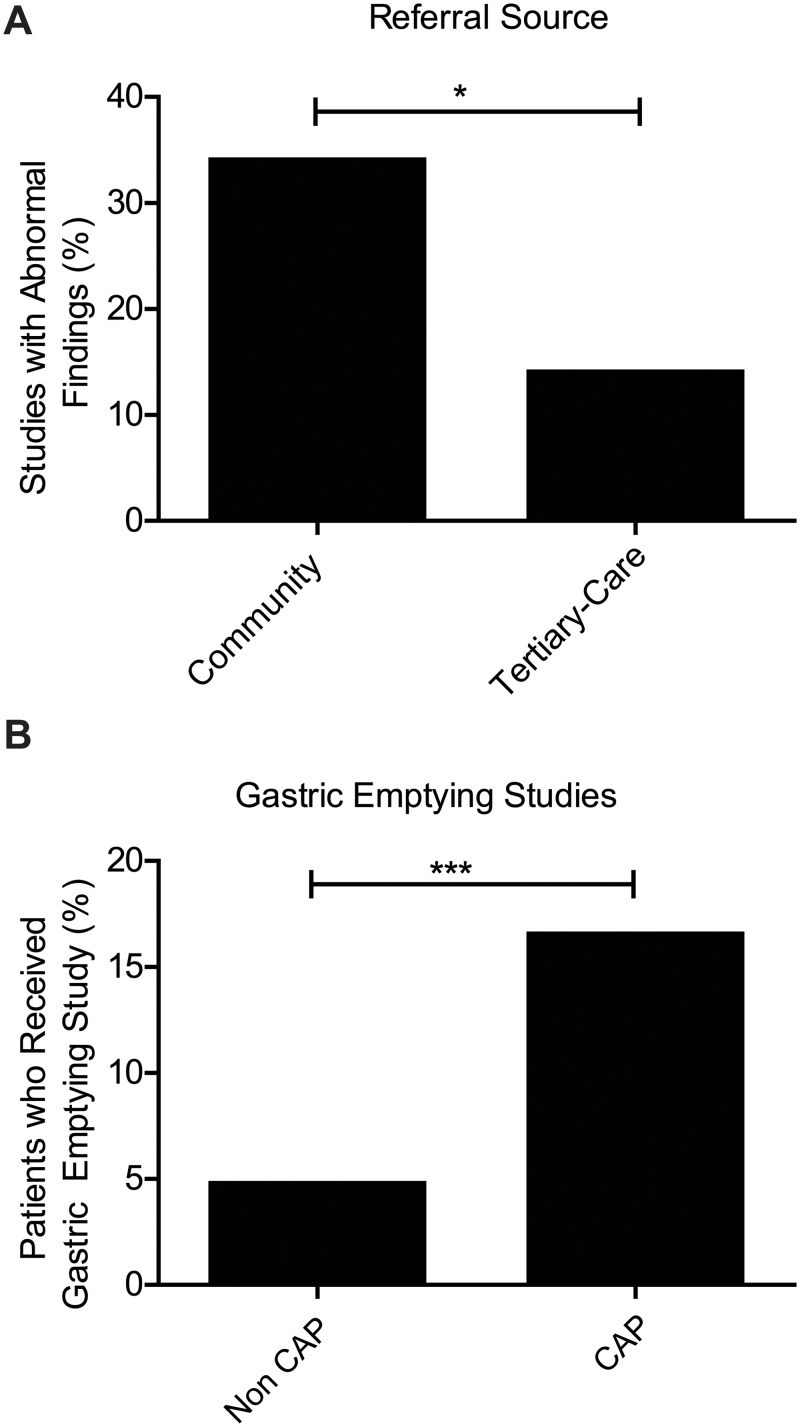
Difference in patients with chronic abdominal pain (CAP). (A) Abnormal findings by referral source. Patients receiving capsule endoscopy for CAP from the community setting had a significantly higher rate of abnormal findings than those referred for capsule studies from a tertiary center. (B) Patients with CAP who also received gastric emptying studies.

Because our retrospective data was collected from an open endoscopy center, we compared patients referred from community physicians (47 patients; 52.2% of patients with CAP) and those from the tertiary care facility (43 patients; 47.8% of patients with CAP). Community physicians referred the majority of patients with CAP and no other symptoms, (20 patients; 64.5% of CAP-O). However, patients referred from community physicians were much more likely to have abnormal findings on VCE studies compared to those from tertiary care facility (34.0% v 14.0%, respectively, p = 0.003) (Fig 3A).

### Additional Testing After VCE

We also investigated the use of other diagnostic procedures for patients with CAP. Interestingly, we found that CAP-O patients were more likely to have had gastric emptying studies performed compared to the control cohort (16.5% v 4.8%, respectively, p = 4.0x10^-5^). However, they were not likely to have abnormal findings from those studies as well, questioning need for such an extensive work-up (Fig 3B). The CAP-A group did not have a significant difference in this particular measure when compared to the control cohort.

### Post-VCE Management and Intervention

In order to further understand the role of VCE in the evaluation of patients with CAP, we investigated whether VCE affected management and interventions. We had follow up data for 61 (67.8%) of patients with CAP, including 18 (58.1%) of CAP-O group and 43 (72.9%) of CAP-A group. Follow up data was available for the vast majority of patients with abnormal findings (16/22; 72.7%) for at least 6 months after VCE. In patients with CAP, 24 out of the 61 (39.3%) had improvement of symptoms on follow-up. The majority, 13 patients (54.2%), were due to spontaneous resolution. Of the CAP group, 11 patients (47.8%) improved from an intervention after VCE. Of the 24 patients whose symptoms resolved after VCE, 19 (79.2%) were from the CAP-A group, 10 (52.6%) of whom benefited from a post-VCE intervention (Table 2, Table 3). The remaining five of the 24 patients were from the CAP-O group, where only one (20%) benefited from an intervention from VCE (Table 4, Table 5). Despite the concern for malignancy, none of the 90 patients evaluated for abdominal pain had masses visualized by VCE.

**Table 2:** Sensitivity analysis of patients with chronic abdominal pain and associated symptoms.

Outcome	CAP-A			Obscure Bleeding of GI tract (VCE-GIB-mA)		
	*Abnormal* VCE	Normal VCE	Total	*Abnormal VCE*	Normal VCE	Total
Spontaneous Resolution	3	6	9	5	23	27
Intervention Related Resolution	7	3	10	24	10	34
Unresolved	3	21	24	6	18	24
Total with Follow Up Data	13	30	43	35	51	86
No Follow Up Data	3	13	16	-	-	-

Sensitivity analysis of patients with chronic abdominal pain and associated symptoms (CAP-A) and 2:1 age- and gender-matched patients who received a VCE for obscure GI Bleeding and had follow up data (VCE-GIB-mA).

**Table 3:** Abnormal findings and outcomes for CAP-A patients.

Indication	Sex	Age	Referral Source	Findings	Location of Finding	Outcome
**CAP + Melena**	F	47	Community	Abnormal Emptying Diffuse erythema	Stomach & Terminal Ileum	CAP Unresolved
**CAP + N/V**	F	63	Community	Patchy erythema	Terminal Ileum	CAP unresolved
**CAP + Anemia**	F	46	Tertiary Center	Ulceration, narrowing	Jejunum & Early Ileum	CAP Unresolved
**CAP + Hematochezia**	F	25	Tertiary Center	Ulcerations	Jejunum & Ileocecal junction	CAP Unresolved
**CAP + Anemia**	F	42	Community	Gastropathy, ulcers, erosions	Stomach & Jejunum	Loss to follow up
**CAP + N/V**	F	32	Tertiary Center	Erythema /duodenitis	Duodenum & Early Jejunum	Loss to follow up
**CAP + Diarrhea**	M	25	Community	Polyps	Distal Ileum	**Resolved after intervention**
**CAP + PMH of Crohn’s**	F	30	Community	Ulceration, nodularity	Jejunum	**Resolved after intervention**
**CAP + PMH of Crohn’s**	F	52	Community	Inflammation, ulceration	Ileocolonic junction	**Resolved after intervention**
**CAP + PMH of Crohn’s**	M	71	Community	Erythema and inflammation	Distal Ileum	**Resolved after intervention**
**CAP + PMH Crohn’s + Weight loss**	M	24	Tertiary Center	Stricture	Jejunum	**Resolved after intervention**
**CAP + PMH of Crohn’s**	M	71	Tertiary Center	Inflamed/ulcerated stricture	Jejunum & Ileum	**Resolved after intervention**
**CAP + Melena**	F	32	Tertiary Center	Ulcers, erythema and inflammation	Stomach & Ileum	**Resolved after intervention**
**CAP + N/V**	F	44	Community	Nodular mucosa and erythema	Jejunum	Spontaneous resolution
**CAP + N/V**	M	26	Community	Erythema	Terminal Ileum	Spontaneous resolution
**CAP + Diarrhea**	M	29	Community	Erythema/edema	Jejunum	Spontaneous resolution

**Table 4:** Sensitivity Analysis for chronic abdominal pain without associated symptoms.

Outcome	CAP-O			Obscure Bleeding of GI tract (VCE-GIB-mO)		
	*Abnormal* VCE	Normal VCE	Total	*Abnormal VCE*	Normal VCE	Total
Spontaneous Resolution	1	3	4	3	11	14
Intervention Related Resolution	1	0	1	10	4	14
Unresolved	1	12	13	2	6	8
Total with Follow Up Data	3	15	18	15	21	36
No Follow Up	3	10	13	-	-	-

Sensitivity analysis of patients with chronic abdominal pain and no other symptoms (CAP-O) and 2:1 age- and gender-matched patients who received a VCE for obscure GI Bleeding who had follow up data (VCE-GIB-mO).

**Table 5:** Abnormal findings and outcomes for chronic abdominal pain (without other symptoms).

Indication	Sex	Age	Referral Source	Findings	Location of Finding	Outcome
**CAP Only**	F	46	Community	Erythematous spots	Jejunum	CAP unresolved
**CAP Only**	F	52	Community	Ulcers	Distal Jejunum	Loss to follow up
**CAP Only**	F	56	Community	White polypoid protrusion (cyst vs polyp vs lymphoid tissue)	Early Ileum	Loss to follow up
**CAP Only**	M	36	Community	Enteritis	Early Jejunum	Loss to follow up
**CAP Only**	M	62	Community	Stricture with ulceration	Proximal ileum	**Resolved after Intervention**
**CAP Only**	F	36	Community	Ulcerations, Erythema	Ileum	Spontaneous resolution

Of the 11 CAP patients who benefitted from an intervention, VCE was abnormal in eight (72.7%). Active Crohn’s disease, enteropathy, and ulcerations were the most common diagnoses following an abnormal study for patients with CAP. Accordingly, the most common interventions based on these diagnoses were implementation of an immune suppression therapy and cessation of NSAID use. Out of the eight cases where VCE led to a change in management, five (62.5%) were due to Crohn’s disease related therapy, in patients with a prior history of the disease (S2 and S3 Tables). In addition, there were three patients who resolved after an intervention, but had a normal VCE. One patient resolved pain with a change in diet. The CAP of the other two resolved with marijuana cessation.

### Sensitivity Analysis

**Fig 4:**
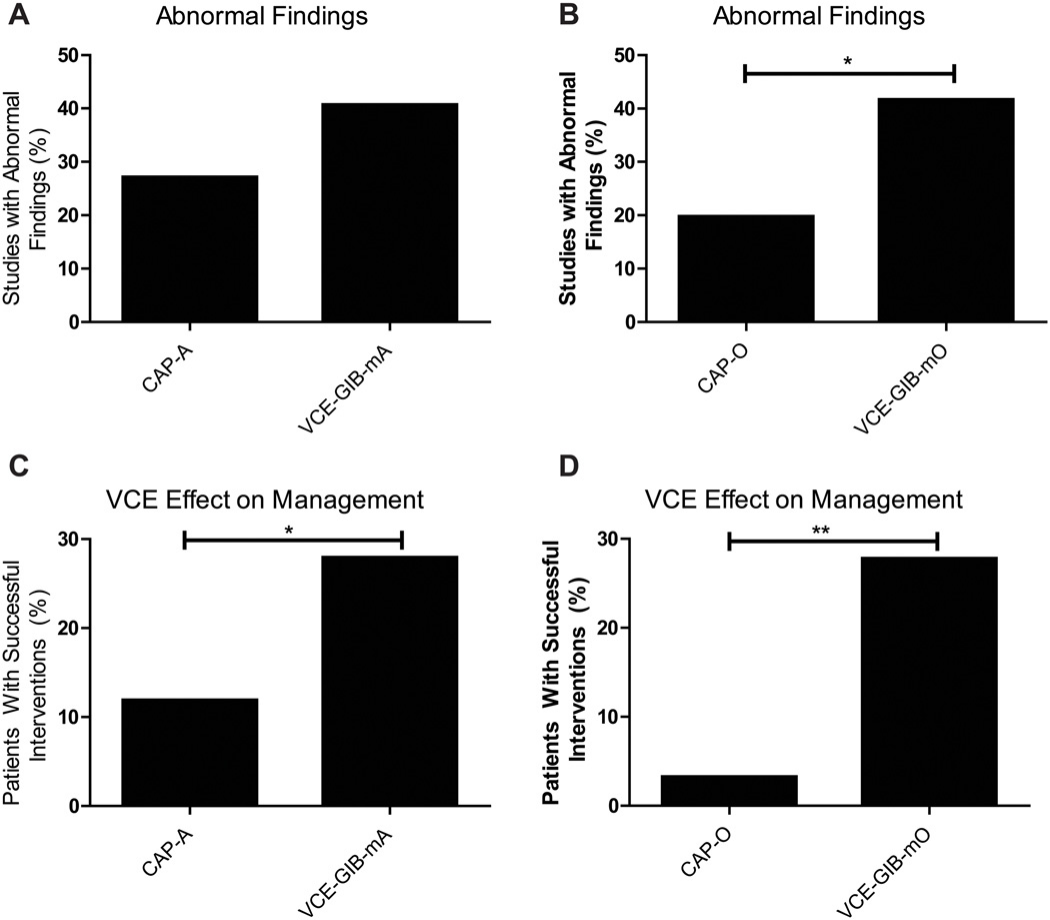
Sensitivity analysis with 2:1 age and gender matched control (CAP). (A) Abnormal finding rates in patients with CAP and no other symptoms compared to matched patients who were evaluated for gastrointestinal bleeding (B) Comparison of the clinical impact of VCE in the CAP-O cohort and its matched gastrointestinal bleed group. (C) Abnormal finding rates in patients with CAP and other symptoms compared to age and gender matched patients who were evaluated for gastrointestinal bleeding. (D) Comparison of the clinical impact of VCE in the CAP-A cohort and its matched gastrointestinal bleed group. * represents a p-value of < 0.05. ** represents a p-value of < 0.01

In order to determine whether the abnormal finding rate and the frequency of interventions that improved outcomes were similar between patients who received VCE for CAP and patients who received VCE for an indication with strong evidence (i.e. obscure gastrointestinal bleed), we performed a sensitivity analysis. We decided to use VCE-GIB population since the evidence is strong that these patients would benefit from a VCE, and its indication clearly stated in society practice guidelines [10]. In the initial analysis, 86 patients who received VCE for obscure gastrointestinal bleed (VCE-GIB-mA) were randomly selected to correspond with the 43 patients we had follow up data for. These patients were age- and gender-matched to the patients in the CAP-A group (see methods for selection process). In the VCE-GIB-mA group, we found an abnormal finding rate that was not significantly different than that of the CAP-A cohort (40.7% v 27.1%, p = 0.32, respectively, Fig 4A). We repeated this analysis for the CAP-O cohort by age- and gender-matching VCE-GIB-mO patients whom we had follow up data for in a 2:1 ratio (total of 36 patients). Fifteen out of the 36 cases (41.7%) had abnormal findings on VCE study. The CAP-O cohort had significantly lower rate of abnormal findings than the VCE-GIB-mO patients (19.7% v 41.7%, p = 0.009, respectively, Fig 4B).

We found differing results when analyzing patients with successful interventions as a result of VCE. Twenty four out of the 86 cases (27.9%) experienced a change in management or intervention due to the abnormal VCE study. This success rate was significantly different to that of CAP-A patients (11.8% v 27.9%, p = 0.03, respectively, Table 2, Fig 4C). Of the 36 patients in the VCE-GIB-mO cohort, 10 out of the 36 cases (27.8%) experienced a change in management or intervention due to the abnormal VCE study. The successful intervention rate for CAP-O was significantly less than that of the matched VCE-GIB-mO cohort (3.2% v 27.8%, p = 0.008, respectively, Table 4, Fig 4D).

Since many of the CAP-A patients who benefited from an intervention subsequent to VCE were Crohn’s disease patients, we decided to analyze them separately. When the Crohn’s disease patients were removed from analysis, there was still no significant difference between CAP-A and VCE-GIB-mA cohorts in percentage with abnormal findings (23.9% v 40.0%, p = 0.12, respectively, S2 Fig). However, the percentage of CAP-A patients with a successful intervention decreased significantly (4.4% v 23.3%, p = 0.004). Finally, we compared patients with CAP and a history of Crohn’s disease to their matched cohort (VCE-GIB-mCD). In patients with CAP and a history of Crohn’s, there was no significant difference in successful intervention (38.5% v 38.4%, CAP with Crohn’s v VCE-GIB-mCD, p = 1.0, respectively, S3 Fig).

## Discussion

Patients with CAP, as a whole, were less likely to have abnormal findings on VCE when compared to those who received VCE for other indications. Importantly, no small bowel malignancies were identified in the CAP group. However, when the patients were subdivided into those who had CAP as their sole symptom (CAP-O) versus those who had associated symptoms (CAP-A), differences started to emerge. VCE rarely was helpful in the CAP-O group. The diagnostic yield of the exam was low. Furthermore, it was unlikely that an abnormal finding would lead to a change in management of the patient’s disease. However, for the CAP-A group, the abnormal finding rate was not different from VCE performed for other indications. Moreover, some patients benefitted since VCE changed management at a rate equivalent compared to VCE performed for gastrointestinal bleeding, which is a well-established indication and part of the AGA and ASGE guidelines for evaluation of obscure gastrointestinal bleeding.

Previous retrospective studies of VCE for CAP have reported various diagnostic rates. However, whether these rates are high or low, or worth the cost of performing the diagnostic test became a matter of debate. Our study contributes to the understanding the role of VCE in CAP in two ways. It first shows whether a higher abnormal finding rate actually translated to clinically meaningful interventions. Furthermore it shows how this rate compares to an age- and gender-matched cohort from the same healthcare system that receives the VCE for a well-established indication. Our study shows that VCE is a useful diagnostic tool that can help provide a possible etiology of CAP in patients with associated symptoms. However, a change in management from information provided by VCE is likely to be limited to those with a history of Crohn’s disease. Despite the presence of strictures and risk of capsule retention, our study did not report any complications from VCE in Crohn’s disease patients. This is consistent with previous literature that shows VCE has significant clinical impact in IBD [11–14].

In our study, VCE yielded abnormal findings in 24.4% of patients with CAP. This rate is consistent with a recent systematic review regarding CAP and VCE, which reported a rate of 20.9% [9]. Out of the 21 studies selected in their review, diagnostic rate varied from 4–44%, with over 12% standard deviation from an unadjusted mean of 22%. The usefulness of VCE in patients with CAP remained dubious due to drastically varying diagnostic yields and other significant limitations in the literature evaluated. This study addresses many of those limitations and also offers a simple classification by which physicians can classify a patient with CAP. It also provides an explanation of why such high variance in diagnostic yield exists since these studies may have had different proportion of CAP-O and CAP-A patients. In CAP-O patients, VCE has a lower diagnostic yield and is much less likely to lead to an intervention that improves outcomes. However, in CAP-A patients, VCE could be a useful tool, providing relevant findings at a rate similar to that of age- and gender-matched patients who were getting VCE for gastrointestinal bleeding. However, even in this group, these findings did not translate to successful interventions. The only group that seemed to benefit were those with Crohn’s disease.

Although the abnormal finding rate and successful intervention percentage may seem low for the VCE-GIB groups, this is likely due to the younger age of patients in this age-matched cohort. Furthermore, as an open referral center, many of the patients with gastrointestinal bleeding had their VCE study performed many months after first referral since patients needed prior insurance approval before the procedure. Previous studies have shown that prolonged time between referral and VCE reduces diagnostic yield in patients with obscure GI bleed [15, 16]. Despite this, we believe our matched gastrointestinal bleeding cohorts remain an adequate measure of comparison on VCE impact, given that our cohorts match the majority of literature regarding outcome and overall clinical impact [16–19].

This study has the largest cohort of patients from the United States, however, other larger studies exist from Asia. The various homogenous populations within Asia have differing prevalence of gastrointestinal disease and infectious disease, which may significantly alter predictors of an abnormal study or the average rate of abnormal findings [20]. Furthermore, significant differences in prevalence and etiology of chronic abdominal pain exist in international states compared to the United States [17, 21]. Despite these differences, the largest study to date (n = 243) reported a yield (23%) quite similar to our own [22]. This prospective study involved nine tertiary institutions in China. The second largest CAP study, a retrospective study from Korea (n = 110) reported a yield of 17.3% [23]. Neither study reported differences in yield based upon clinical presentation nor whether VCEs changed management or improved outcomes. The larger studies were primarily prospectively evaluating the usefulness of VCE at their respective institutions, all of which were tertiary referral centers. This study is the first to report differences in CAP management and treatment following VCE.

Smaller studies have reported a much greater variation in yield. A multicenter study from Greece (n = 72) reported an overall diagnostic yield of 44.4% for CAP, while another study (n = 64) reported an overall diagnostic yield of 9% [24, 25]. Other studies have reported a diagnostic yield of 36–40% (n = 50) and 6.3% (n = 16) [26, 27]. Hence, our studies abnormal finding yield falls within the large range reported in previous literature. Moreover, it confirms previous reports that the diagnostic yield of VCE improves when CAP is present with other associated symptoms [23–25].

Over the last several years, enterography studies of the small bowel (either by magnetic resonance [MR] or by computed tomography [CT]) have increasingly been used as diagnostic tools in the evaluation of patients with suspected or established IBD [28, 29]. A significant portion of our studies were performed prior to this trend and enterography is a commonly used alternative to VCE in patients with IBD. Whether they are superior to VCE in diagnosis is debatable and outside the scope of this study [30–35]. The role of enterography and any potential advantages in its evaluation of CAP remains unclear. Meanwhile, VCE has proven a cost effective, highly sensitive, and clinically useful modality in the evaluation of IBD that offers a diagnostic advantage in mild to moderate disease activity in Crohn’s disease where mild tissue inflammation and shallow ulcerations may not be obvious on enterography [32, 34, 36–39].

The rate of abnormal findings in this study is similar to that of other diagnostic modalities utilized the evaluation of CAP as well. Colonoscopy and esophagogastroduodenoscopy (EGD) have been reported to have a diagnostic yield of 24%, and 23–46%, respectively. [40, 41]. Unlike EGDs and colonoscopies where ancillary findings such as abnormal imaging and/or serum biomarkers increase the incidence of abnormal findings, yield of VCE in patients with CAP did not improve with the incorporation of these factors. Abnormal blood biomarkers such as ESR, CRP, ANA, and leukocytosis were not associated with study abnormalities in any patient subset. Hence, no serum biomarker measures that were helpful in identifying a subgroup of patients with CAP who may benefit from VCE.

Community physicians were referring the majority of the CAP-O population. Although we presumed that those in our tertiary care center were more likely to have abnormal findings due to the complexity of their cases, community gastroenterologists were more likely to refer patients that would eventually have an abnormal study. Furthermore, the increased of use of gastric emptying study in the patients in the CAP-O group further confirms the suspicion that there is an over utility of diagnostic tests in the CAP population.

VCE is a low risk diagnostic modality for visualizing the gastrointestinal tract. As such, it is increasingly being utilized for patients with potential abdominal sources of disease. However, the studies are expensive, require skilled monitoring and review, and present some risk for complications associated with capsule retention. Although none of our patients with CAP had a complication from VCE, capsules can rarely cause an obstruction requiring a surgical intervention. CAP without other associated symptoms appears to be a low yield indication for VCE given a low rate of abnormal findings, and lower likelihood that an abnormal finding could lead to an intervention. In addition, no malignancies of the small bowel were identified. More than a third of patients with CAP had no associated symptoms. This finding is particularly useful for healthcare costs. However, larger, multi-center studies would still be helpful to confidently evaluate whether VCE is consistently helpful in patients with associated symptoms or a worrisome past medical history.

While this large retrospective study sampled a diverse population of patients from throughout San Diego and Imperial Counties, it has its limitations. We did not have follow up data for all of the patients who received VCE for CAP. Furthermore, chart review was limited by the completeness of physician documentation and did not allow for interpretation of intent, unless explicitly stated. Hence, we were unable to quantify any benefit of a negative study. Also, we acknowledge the inability of this study to comment on the characteristics of patients who had abdominal pain, but were not referred to the UCSD endoscopy center for a VCE. Such patients may represent those with less impressive clinical presentations or lower pre-test probability of disease.

In conclusion, the role of VCE in CAP remains complex. Although VCE is unlikely to be beneficial in patients without associated symptoms, it may play a role in those who have associated symptoms. In this study, VCE not only had an abnormal finding rate that was comparable to upper and lower endoscopy, but also was likely to lead to altered disease management. More prospective studies are needed to better understand the role that VCE can play in CAP.

## Supporting Information

S1 Fig Flowchart with classification of patients from the control group; patients evaluated for reasons other than chronic abdominal pain group. Sub-group classification based on similar presentation. Abbreviations: GI (gastrointestinal), HHT (heredity hemorrhagic telangiectasia), Hx (history), FAP (familial adenomatous polyposis).
(DOCX)

S2 Fig Abnormal Finding Rate if Crohn’s Disease Patients Are Excluded. Abnormal finding rate comparison between patients with CAP-A, but excluding patients with a history of Crohn’s disease (CAP-A without CD) and their 2:1 age- and gender-matched comparison group as previously described, without Crohn’s disease matched patients (VCE-GIB-mA^-CD^).
(DOCX)

S3 Fig Inclusion and Exclusion of Crohn’s Disease Patients on Treatment Outcomes. CAP with CD, CAP-A without CD, and their 2:1 age, gender matched control group (VCE-GIB-mCD, VCE-GIB-mA^-CD^, respectively). ** represents a p-value of < 0.01.
(DOCX)

S1 Table Comparison of Inflammatory markers (not available for many patients) versus abnormal findings. Abbreviations: ESR: Erythrocyte sedimentation rate. CRP: C Reactive Protein
(DOCX)

S2 Table Abnormal findings and outcomes for patients with chronic abdominal pain (CAP) and Crohn’s disease. (DOCX)

S3 Table Sensitivity analysis of patients with Crohn’s Disease (CD) and chronic abdominal pain (CA) compared to both patients with CAP and associated symptoms without Crohn’s Disease (CAP-A without CD), the 2:1 gender and aged matched patients for CAP-A without CD in patients who received a VCE for gastrointestinal bleeding and had follow up data (VCE-GIB-mA*). (DOCX)
