# Supplementary material for: Video Capsule Endoscopy in Patients with Chronic Abdominal Pain with or without Associated Symptoms: A Retrospective Study
Source: PLoS One. 2015 Apr 20;10(4):e0126509. doi: 10.1371/journal.pone.0126509 (PMC4404061; doi:10.1371/journal.pone.0126509)
Supplement: S1 Fig — Sub-group classification based on similar presentation. Abbreviations: GI (gastrointestinal), HHT (heredity hemorrhagic telangiectasia), Hx (history), FAP (familial adenomatous polyposis). (DOCX) [file pone.0126509.s001.docx]

**S1 Figure:** Flowchart with classification of patients from the control group; patients evaluated for reasons other than chronic abdominal pain group. Sub-group classification based on similar presentation. Abbreviations: GI (gastrointestinal), HHT (heredity hemorrhagic telangiectasia), Hx (history), FAP (familial adenomatous polyposis).
