# Supplementary material for: Video Capsule Endoscopy in Patients with Chronic Abdominal Pain with or without Associated Symptoms: A Retrospective Study
Source: PLoS One. 2015 Apr 20;10(4):e0126509. doi: 10.1371/journal.pone.0126509 (PMC4404061; doi:10.1371/journal.pone.0126509)
Supplement: S2 Fig — Abnormal finding rate comparison between patients with CAP-A, but excluding patients with a history of Crohn’s disease (CAP-A without CD) and their 2:1 age- and gender-matched comparison group as previously described, without Crohn’s disease matched patients (VCE-GIB-mA-CD). (DOCX) [file pone.0126509.s002.docx]

**S2 Figure:** Abnormal Finding rate : Analysis excluding patients with Crohn’s Disease. Comparison between patients with Chronic Abdominal Pain and associated symptoms, but no history of Crohn’s Disease (CAP-A without CD) and its 2:1 age and gender matched comparison group as previously described, without CD matched patients (VCE-GIB-mA*).
