# Supplementary material for: Video Capsule Endoscopy in Patients with Chronic Abdominal Pain with or without Associated Symptoms: A Retrospective Study
Source: PLoS One. 2015 Apr 20;10(4):e0126509. doi: 10.1371/journal.pone.0126509 (PMC4404061; doi:10.1371/journal.pone.0126509)
Supplement: S3 Fig — CAP with CD, CAP-A without CD, and their 2:1 age, gender matched control group (VCE-GIB-mCD, VCE-GIB-mA-CD, respectively). ** represents a p-value of < 0.01. (DOCX) [file pone.0126509.s003.docx]

**S3 Figure:** VCE effect on Management by all subgroups. Analysis excluding patients with Crohn’s Disease.; CAP and CD, VCE-GIB-mA, CAP-A without CD, patients with chronic abdominal pain but no other symptoms (CAP-O), and their 2:1 age, gender matched control group (VCE-GIB-mO). * represents a p-value of < 0.05. ** represents a p-value of < 0.01
