# Supplementary material for: Video Capsule Endoscopy in Patients with Chronic Abdominal Pain with or without Associated Symptoms: A Retrospective Study
Source: PLoS One. 2015 Apr 20;10(4):e0126509. doi: 10.1371/journal.pone.0126509 (PMC4404061; doi:10.1371/journal.pone.0126509)
Supplement: S1 Table — Abbreviations: ESR: Erythrocyte sedimentation rate. CRP: C Reactive Protein (DOCX) [file pone.0126509.s004.docx]

| Inflammatory Markers | Negative | Positive | P value |
| --- | --- | --- | --- |
| ESR | 12 | 11 | 0.25 |
| CRP | 29 | 20 | 0.52 |
| Leukocytosis | 65 | 2 | 0.3 |
| Anti-Nucleic Antibody | 19 | 7 | 0.94 |

**S1 Table:** Comparison of Inflammatory markers (not available for many patients) versus abnormal findings. Abbreviations: ESR: Erythrocyte sedimentation rate. CRP: C Reactive Protein
