# Supplementary material for: Video Capsule Endoscopy in Patients with Chronic Abdominal Pain with or without Associated Symptoms: A Retrospective Study
Source: PLoS One. 2015 Apr 20;10(4):e0126509. doi: 10.1371/journal.pone.0126509 (PMC4404061; doi:10.1371/journal.pone.0126509)
Supplement: S2 Table — (DOCX) [file pone.0126509.s005.docx]

| Indication | Sex | Age | Referral Source | Findings | Outcome |
| --- | --- | --- | --- | --- | --- |
| CAP + PMH of Crohn’s | M | 29 | Community | No Abnormal Finding | CAP Unresolved |
| CAP + PMH of Crohn’s | M | 71 | Community | Erythema and inflammation | **Resolved after intervention** |
| CAP + PMH of Crohn’s | M | 71 | Tertiary Center | Inflamed/ulcerated stricture | **Resolved after intervention** |
| CAP + PMH of Crohn’s | M | 26 | Community | No Abnormal Finding | CAP Unresolved |
| CAP + PMH of Crohn’s + Weight Loss | M | 24 | Tertiary Center | Stricture | **Resolved after intervention** |
| CAP + PMH of Crohn’s | F | 52 | Community | Inflammation, ulceration | **Resolved after intervention** |
| CAP + PMH of Crohn’s | F | 30 | Community | Ulceration, nodularity | **Resolved after intervention** |
| CAP + PMH of Crohn’s | F | 25 | Tertiary Center | No Abnormal Finding | CAP Unresolved |
| CAP + PMH of Crohn’s | M | 25 | Community | No Abnormal Finding | Spontaneous resolution |
| CAP + PMH of Crohn’s | F | 47 | Community | No Abnormal Finding | Spontaneous resolution |
| CAP + PMH of Crohn’s | M | 23 | Community | No Abnormal Finding | CAP Unresolved |
| CAP + PMH of Crohn’s | F | 19 | Tertiary Center | No Abnormal Finding | CAP Unresolved |
| CAP + PMH of Crohn’s | F | 23 | Tertiary Center | No Abnormal Finding | Spontaneous resolution |

**S2 Table:** Abnormal findings and outcomes for patients with chronic abdominal pain (CAP) and Crohn’s disease.
