# Supplementary material for: Video Capsule Endoscopy in Patients with Chronic Abdominal Pain with or without Associated Symptoms: A Retrospective Study
Source: PLoS One. 2015 Apr 20;10(4):e0126509. doi: 10.1371/journal.pone.0126509 (PMC4404061; doi:10.1371/journal.pone.0126509)
Supplement: S3 Table — (DOCX) [file pone.0126509.s006.docx]

| Outcome | CAP-A without CD | | | VCE-GIB-mA* | | | CAP and CD | | | |
| --- | --- | --- | --- | --- | --- | --- | --- | --- | --- | --- |
|  | Abnormal VCE | Normal VCE | Total | Abnormal VCE | Normal VCE | Total | Abnormal VCE | | Normal VCE | Total |
| Spontaneous resolution | 3 | 3 | 6 | 2 | 16 | 18 | 0 | 3 | | 3 |
| Intervention-related resolution | 2 | 3 | 5 | 14 | 10 | 24 | 5 | 0 | | 5 |
| Unresolved | 3 | 16 | 19 | 3 | 15 | 18 | 0 | 5 | | 5 |
| No follow up data | 3 | 13 | 16 | - | - | - | - | - | | - |
| Total with Follow up Data | 8 | 22 | 30 | 19 | 41 | 60 | 5 | 8 | | 13 |

**S3 Table:** Sensitivity analysis of patients with Crohn’s Disease (CD) and chronic abdominal pain (CA) compared to both patients with CAP and associated symptoms without Crohn’s Disease (CAP-A without CD), the 2:1 gender and aged matched patients for CAP-A without CD in patients who received a VCE for gastrointestinal bleeding and had follow up data (VCE-GIB-mA*).
